# Effectiveness of Interferon Beta 1a, compared to Interferon Beta 1b and the usual therapeutic regimen to treat adults with moderate to severe COVID-19: structured summary of a study protocol for a randomized controlled trial

**DOI:** 10.1186/s13063-020-04382-3

**Published:** 2020-06-03

**Authors:** Seyed Sina Naghibi Irvani, Maryam Golmohammadi, Mohamad Amin Pourhoseingholi, Shervin Shokouhi, Ilad Alavi Darazam

**Affiliations:** Department of Infectious Diseases, Loghman Hakim Hospital, Shahid Beheshti University of Medical Sciences, Tehran, Iran

**Keywords:** COVID-19, SARS-COV-2, Randomized controlled trial, Hydroxychloroquine + Lopinavir/Ritonavir, Interferon-β 1a, Interferon-β 1b

## Abstract

**Objectives:**

We will investigate the effectiveness of Interferon Beta 1a, compared to Interferon Beta 1b and the usual therapeutic regimen in COVID-19 in patients that have tested positive and are moderately to severely ill.

**Trial design:**

This is a single center, open label, randomized, controlled, parallel group, clinical trial that will be conducted at Loghman Hakim Medical Education Center in conjunction with Shahid Beheshti University of Medical Sciences.

**Participants:**

Sixty COVID-19 confirmed cases (using the RT-PCR test) will be enrolled in the trial between April 9^th^ to April 14^th^ 2020. Patients will be randomly assigned to the intervention groups or the control group with the following eligibility criteria: ≥ 18 years of age AND (oxygen saturation (SPO2) ≤ 93% OR respiratory rate ≥ 24) AND at least one of the following: Contactless infrared forehead thermometer temperature of ≥37.8, cough, sputum production, nasal discharge, myalgia, headache or fatigue on admission, and time of onset of the symptoms should be acute (Days ≤ 14). Although Hydroxychloroquine will be administered in a single dose, patients with heart problems (prolonged QT or PR intervals, second- or third-degree heart block, and arrhythmias including torsade de pointes) will be excluded. Other exclusion criteria include using drugs with potential interaction with Hydroxychloroquine + Lopinavir/Ritonavir, Interferon-β 1a, Interferon-β 1b, pregnant or lactating women, history of alcohol or drug addiction in the past 5 years, blood ALT/AST levels > 5 times the upper limit of normal on laboratory results and refusal to participate.

This study will be undertaken at the Loghman Hakim Hospital, Shahid Beheshti University of Medical Sciences and Health Services.

**Intervention and comparator:**

COVID-19 confirmed patients will be randomly assigned to one of three groups, with 20 patients in each. The first group (Arm 1) will receive Hydroxychloroquine + Lopinavir / Ritonavir (Kaletra) + Interferon-β 1a (Recigen), the second group (Arm 2) will be administered Hydroxychloroquine + Lopinavir / Ritonavir (Kaletra) + Interferon-β 1b (Ziferon), and the control group (Arm 3) will be treated by Hydroxychloroquine + Lopinavir / Ritonavir (Kaletra).

**Main outcomes:**

Time to clinical improvement is our primary outcome measure. This is an improvement of two points on a seven-category ordinal scale (recommended by the World Health Organization: Coronavirus disease (COVID-2019) R&D. Geneva: World Health Organization) or discharge from the hospital, whichever comes first.

Secondary outcomes include mortality from the date of randomization until the last day of the study which will be the day all of the patients have had at least one of the following outcomes: 1) Improvement of two points on a seven-category ordinal scale. 2) Discharge from the hospital 3) Death. If any patient dies, we have reached an important secondary outcome. SpO2 Improvement between the last and first day of hospitalization, using pulse-oximetry. Duration of hospitalization from date of randomization until the date of hospital discharge or date of death from any cause, whichever comes first. Incidence of new mechanical ventilation uses from date of randomization until the last day of the study. Please note that we are trying to add further secondary outcomes and this section of the protocol is still evolving.

Statistical analysis will be performed by R version 3.6.1 software. We will use Kaplan–Meier to analyze the time to clinical improvement (compared with a log-rank test). Hazard ratios with 95% confidence intervals will be calculated using the Cox proportional-hazards model in crude and adjusted analysis.

**Randomization:**

Eligible patients will be randomly assigned in a 1:1:1 ratio to receive either Interferon Beta 1a, Interferon Beta 1b or standard care only. Patients will be randomly allocated to three therapeutic arms using permuted, block-randomization to balance the number of patients allocated to each group. The permuted block (three or six patients per block) randomization sequence will be generated, using Package ‘randomizeR’ in R software version 3.6.1. and placed in individual sealed and opaque envelopes by the statistician. The investigator will enroll the patients and only then open envelopes to assign patients to the different treatment groups. This method of allocation concealment will result in minimum selection and confounding biases.

**Blinding (Masking):**

The present research is open-label (no masking) of patients and health care professionals who are undertaking outcome assessment of the primary outcome - time to clinical improvement.

**Numbers to be Randomized (Sample Size):**

Of the 60 patients who underwent randomization, 20 patients were assigned to receive Interferon beta-1a, 20 patients were assigned to receive Interferon beta 1b plus standard care and the rest of patients were assigned to receive the standard care alone.

**Trial Status:**

Protocol version 1.2.1. Recruitment is finished, the start date of recruitment was on 9^th^ April 2020 and the end date was on 14^th^ April 2020. Last point of data collection will be the last day on which all of the 60 participants have had an outcome of clinical improvement or death, completing the study’s follow-up time window.

**Trial registration:**

This study was registered with National Institutes of Health Clinical trials (www.clinicaltrials.gov; identification number NCT04343768, registered April 8, 2020 and first available online April 13, 2020).

**Full protocol:**

The full protocol is attached as an additional file, accessible from the Trials website (Additional file 1). In the interest in expediting dissemination of this material, the familiar formatting has been eliminated; this Letter serves as a summary of the key elements of the full protocol.

## Supplementary information

**Additional file 1.** Full study protocol.

## Data Availability

All the authors will have access to the final trial dataset. Furthermore, the data set the data will be available from the author on reasonable request (Contact: sina.irvani@gmail.com)

