# Supplementary material for: Effectiveness of Interferon Beta 1a, compared to Interferon Beta 1b and the usual therapeutic regimen to treat adults with moderate to severe COVID-19: structured summary of a study protocol for a randomized controlled trial
Source: Trials. 2020 Jun 3;21:473. doi: 10.1186/s13063-020-04382-3 (PMC7268172; doi:10.1186/s13063-020-04382-3)
Supplement: Supplementary file 1 — Additional file 1. Full study protocol. [file 13063_2020_4382_MOESM1_ESM.docx]

**Protocol**

This trial protocol has been provided by the authors to give readers additional information about their work.

**Effectiveness of Interferon Beta 1a and Interferon Beta 1b compared to the Usual Therapeutic Regimen to Treat Adults with Moderate to Severe COVID-19**

A single center, open label, randomized, controlled, parallel group, clinical trial

**Clinical Study Protocol**

**Trial Code:** NCT04343768

**Sponsored by:** Shahid Beheshti University of Medical Sciences

**Study Phase:** Phase II

**Indication for Present Study:** The current outbreak of COVID19 and difficulties caused by the COVID-19 pandemic

**Trial Protocol Authors:**

Dr. Seyed Sina Naghibi Irvani, MD, MPH, MBA

Dr. Maryam Golmohammadi, MD

Dr. Mohamad Amin Pourhoseingholi, PhD

Dr. Ilad Alavi Darazam, MD

Dr. Shervin Shokouhi, MD

**Coordinating Center:** Loghman Hakim Hospital

**TABLE OF CONTENTS**

PROTOCOL ENGLISH SUMMARY……………………………………………….....................................4

1. INTRODUCTION……………………………………………….……………………………………...7
   1. Background………………………...................................................................................................7
   2. Research History…………………...…............................................................................................8
2. STUDY OBJECTIVES…………………………………………………………………….…………...9

2.1 Primary Objective.……………………………..................................................................................9

2.2 Secondary Objectives……………………….....................................................................................9

2.3 Practical Objective…………………………………………….…………………………………...10

2.4 Hypothesis…………………………………………………….…………………………………...10

1. STUDY DESIGN……………………………………………….………………………………...…...11

3.1 General…………………………………………….........................................................................11

3.2 Efficacy Assessments………………………………………….……………………..................…12

3.2.1 Study Outcomes…………………………………………………………….………………12

3.3 Study Drugs………………..............................................................................................................13

3.4 Statistical Methods……………………………………….……………………………..............…13

1. STUDY POPULATION…………………………………………………………………...............….13
   1. Participants…………………………………..................................................................................13
   2. Inclusion Criteria……………………….....................................................................................…14
   3. Exclusion Criteria………………………………………….…..................................................….15
   4. Drop out……………………………………...........................................................................……15
2. RESTRICTIONS………………….....................................................................................................16
3. ETHICAL CONSIDERATIONS………….………………….……………………….....................…16
4. REFERENCES……………………………………………….………..............................................…17

**PROTOCOL ENGLISH SUMMARY**

**Study Title:**

Effectiveness of Interferon Beta 1a and Interferon Beta 1b compared to the Usual Therapeutic Regimen to Treat Adults with Moderate to Severe COVID-19

**Objectives:**

We will investigate the effectiveness of Interferon Beta 1a, compared to Interferon Beta 1b and the usual therapeutic regimen in COVID-19 in patients that have tested positive and are moderately to severely ill.

**Trial Design:**

This is a single center, open label, randomized, controlled, parallel group, clinical trial that will be conducted at Loghman Hakim hospital in conjunction with Shahid Beheshti University of Medical Sciences.

**Participants:**

Sixty COVID-19 confirmed cases (using the RT-PCR test) will be enrolled in the trial between April 9^th^ to April 14^th^ 2020. Patients will be randomly assigned to the intervention groups or the control group with the following eligibility criteria: ≥ 18 years of age AND (oxygen saturation (SPO2) ≤ 93% OR respiratory rate ≥ 24) AND at least one of the following: Contactless infrared forehead thermometer temperature of ≥37.8°C, cough, sore throat, nasal congestion/drip, myalgia, headache or fatigue on admission, and onset of the symptoms should be acute (Days ≤ 14). Although Hydroxychloroquine will be administered in a single dose, patients with heart problems (prolonged corrected QT (450 milliseconds) or PR intervals, second- or third-degree heart block, and history of arrhythmias including torsade de pointes) will be excluded. Other exclusion criteria include using drugs with potential interaction with Hydroxychloroquine + Lopinavir/Ritonavir, Interferon-β 1a, Interferon-β 1b, pregnant or lactating women, history of alcohol or drug addiction in the past 5 years, blood ALT/AST levels > 5 times the upper limit of normal on laboratory results and refusal to participate.

This study will be undertaken at the Loghman Hakim Hospital, Shahid Beheshti University of Medical Sciences.

**Intervention and Comparator:**

COVID-19 confirmed patients will be randomly assigned to one of three groups, with 20 patients in each. The first group (Arm 1) will receive Hydroxychloroquine + Lopinavir / Ritonavir (Kaletra) + Interferon-β 1a (Recigen), the second group (Arm 2) will be administered Hydroxychloroquine + Lopinavir / Ritonavir (Kaletra) + Interferon-β 1b (Ziferon), and the control group (Arm 3) will be treated by Hydroxychloroquine + Lopinavir / Ritonavir (Kaletra).

**Efficacy Assessments:**

**Primary outcome:**

Time to clinical improvement is our primary outcome measure. This is an improvement of two points on a seven-category ordinal scale (recommended by the World Health Organization: Coronavirus disease (COVID-2019) R&D. Geneva: World Health Organization) or discharge from the hospital, whichever comes first.

**Secondary outcomes:**

Secondary outcomes include mortality from the date of randomization until the last day of the study which will be the day all of the patients have had at least one of the following outcomes: 1) Improvement of two points on a seven-category ordinal scale. 2) Discharge from the hospital 3) Death. If any patient dies, we have reached an important secondary outcome. SpO2 Improvement between the last and first day of hospitalization, using pulse-oximetry. Duration of hospitalization from date of randomization until the date of hospital discharge or date of death from any cause, whichever comes first. Incidence of new mechanical ventilation uses from date of randomization until the last day of the study. Please note that we are trying to add further secondary outcomes and this section of the protocol is still evolving.

**Statistical Methods:**

Statistical analysis will be performed by R version 3.6.1 software. We will use Kaplan–Meier to analyze the time to clinical improvement (compared with a log-rank test). Hazard ratios with 95% confidence intervals will be calculated using the Cox proportional-hazards model in crude and adjusted analysis.

1. **مقدمه (Introduction)**
   1. **بیان مسئله (Background)**

تا اول مارس 2020 ، تست کرونا ویروس (COVID-19) 81116 بیمار در چین و 110011بیمار درخارج ازچین مثبت بوده است. در میان بیماران چینی ، تعداد موارد مرگ و میر 3231 مورد بوده است که معادل نرخ مرگ و میر 3.98% است. در حالی که تعداد موارد کشته شده بر اثر COVID-19 در خارج از چین 4576 مورد گزارش شده است. تا همین تاریخ نیز در ایران تست کرونا ویروس 16169 نفر مثبت گزارش شده است و تعداد موارد مرگ و میر 988 نفر بوده است که طبق این گزارش نرخ مرگ و میر در ایران 6.11% بوده است (1).

Coronavirus SARS-CoV-2 یک ویروس RNA تک رشته ای است که مسئول بیماری تنفسی شدید و عفونت شبیه پنومونی در سراسر جهان می باشد که امروزه مورد توجه قرار گرفته است و تبدیل به یک اورژانس بهداشت جهانی شده است(2). SARS-CoV-2 متعلق به خوشه β-coronavirus، همانند SARS-CoV و MERS-CoV است (3). آلوده شدن سلول های پستانداران با ویروس مذکور باعث می شود سیستم ایمنی ذاتی بدن برای ایجاد اولین خط دفاعی فعال شود. اینترفرون ها (IFN) در این رویداد نقش اساسی ایفا می کنند؛ زیرا سیستم ایمنی ذاتی را فعال کرده و به ایمنی اکتسابی نیز کمک می کنند. دو نوع IFN در ایجاد خاصیت ضد ویروسی دخیل هستند: نوع I اینترفرون که توسط اکثر انواع سلول به عنوان پاسخ مستقیم به ویروس سنتز می شود، در حالی که نوع II اینترفرون پس از تماس با سلولهای حاوی آنتی ژن توسط سلولهای ایمنی بدن تولید می شود. هر دو نوع I و نوع II خاصیت ضد ویروسی، ضد پرولیفراتیو و متعادل کننده سیستم ایمنی را دارا هستند(4). در یک مدل موشی که مبتلا به عفونت SARS- CoV بود، عدم تعادلIFN نوع I و سلولهای التهابی به عنوان اصلی ترین علت پنومونی کشنده نشان داده شد(5). شناخت الگوی القای سیستم ایمنی در بزرگسالان و کودکان در سندرم های تنفسی ناشی ازCoV می تواند به یافتن راهکارهای درمانی برای این بیماری کشنده کمک کند.

با توجه به کمبود اطلاعات موجود در مورد COVID-1، SARS می تواند الگوی مفیدی در این زمینه باش؛زیرا SARS-CoV-2 بیشترین شباهت در ساختار و توالی نوکلئوتیدی با SARS-CoV در بین سایر ویروس های این خانواده نشان می دهد (6). در یک مطالعه نیز که در همین زمینه انجام شده بود، متوجه شدند که به نظر می رسد که coronavirus مهمترین پروتئین های موجود در مسیر سیگنالینگ IFN را هدف قرار می دهد تا بتواند با سیستم ایمنی بدن مقابله کند. همین جاست که نقش اصلی آنتی ویروس با واسطه IFN را در پاسخ به عفونت CoV برجسته می کند (7). در حال حاضر IFN-β 1a برای انواع مختلفی از بیماری ها از جمله مولتیپل اسکلروز مورد استفاده قرار می گیرد و اثربخشی آن در چندین کارآزمایی بالینی وابسته به دوز نشان داده است. به طور قابل توجهی ، IFN-β 1a در دوز هایی که در مطالاعات پیشین نشان داده شده است فعالیت ضد ویروسی قوی را داراست و همچنین دارای پروفایل ایمنی قابل قبولی نیز هست. در یک مطالعه که در سال 2003 انجام شده بود، به بررسی درمان SARS با اینترفرون های انسانی پرداختند و متوجه شدند که IFN-β به میزان 5 تا 10 برابر موثرتر از بقیه ی انواع اینترفرون ها بوده است و بهترین داروی ضد ویروسی در مقابله با SARS-CoV بوده است (8).

با توجه به مطالب ذکر شده، ما نیز بر آن شدیم تا به بررسی اثر درمانی هدفمند داروی اینترفرون بتا یک آ در مقایسه با اینترفرون بتا یک ب و نیز رژیم درمانی رایج در بیماران مبتلا به کووید-19 متوسط تا شدید بپردازیم.

- 1. **بررسی متون (Research History)**

کوماری و همکاران(2020) یک مطالعه تحت عنوان COVID-19 به دارویIFN- I حساس می باشد انجام دادند.در این مطالعه SARS-CoV-2 USA-WA1 / 2020 که توسط مرکز مرجع جهانی ویروسها و اربوویروسها(WRCEVA) آماده شده بود از ایالات متحده آمریکا خریداری شد و سپس پس از ایجاد بیماری در موش ها و استفاده از این دارو،در بررسی های اولیه متوجه شدند که القای زودرس با نوع I IFN می تواند در موش محافظ کننده باشد. به طور کلی ، داده های حساسیت نشان می دهد که نوع I IFN در صورت تعیین پارامترهای مناسب ، ممکن است برای درمان SARS-CoV-2

مفید باشد. علاوه بر این ، استفاده از نوع III IFN ، که پیش بینی می شود در دستگاه تنفسی کارایی داشته باشد ، می تواند وسیله دیگری برای درمان مؤثر برای SARS-CoV-2 باشد.در نهایت متوجه شدند که SARS-CoV-2 حساسیت بسیار بالاتری نسبت به نوع I IFN نسبت به SARS-CoV قبلی دارد. این حساسیت IFN نوع I به احتمال زیاد به دلیل تغییر در پروتئین های ویروسی بین دو سویه CoV اپیدمیک است(9).

لیزا هنسلی و همکاران(2004) یک مطالعه تحت عنوان Interferon-β 1a و تکثیر SARS انجام دادند. در این مطالعه سلول های Vero E-6 داروی Interferon-β 1a در دوز های 5,000 تا 500,000 IU/mLرا 24 ساعت قبل یا 1 ساعت بعد از تلقیح (SARS-CoV (m.o.i. 0.1 PFU/cell دریافت کردند و در 24،48 و 72 ساعت بعد از عفونت بررسی شدند. در بررسی های اولیه متوجه شدند که مهار این ویروس به زمان استفاده دارو و زمان تلقیح عفونت وابسته بود و پیشرفت SARS-CoV به طور بالقوه بعد از 24 ساعت مهار شده بود(≥99.5% یا 2.00 log10 PFU/mL).همچنین متوجه شدند اثربخشی Interferon-β 1a در مواردی که قبل از تلقیح عفونت داده شده بودند،مقداری بیشتر از مواردی بود که بعد از تلقیح عفونت داده شده بودند. با این حال ، تولید SARS-CoVs به طور قابل توجهی در 24 و 48 ساعت پس از ایجاد عفونت کاهش یافته بود (≥ 90 or یا 1.00 log10 PFU / میلی لیتر). در نهایت نیز متوجه شدند که این عفونت پاسخ دراماتیک به این دارو داده بود(10).

1. **اهداف مطالعه (Study Objectives):**

**2.1 هدف اصلی (Primary objective):**

**بررسی اثر درمانی هدفمند داروی اینترفرون بتا یک آ و اینترفرون بتا یک ب در مقایسه با رژیم درمانی رایج در بیماران مبتلا به کووید-19 متوسط تا شدید**

- 1. **اهداف فرعی (Secondary objectives):**
- تعیین و مقایسه فراوانی عوارض جانبی در هريك از 3 گروه درمانی ذکر شده در قسمت روش انجام کار.
- تعیین و مقایسه میانگین سطح اشباع اکسیژن (Sat O2)در هريك از 3 گروه درمانی.
- تعیین و مقایسه میانگین نرخ تنفسی(RR) در هريك از 3 گروه درمانی.
- تعیین و مقایسه میانگین مدت زمان بستری در بیمارستان در هريك از 3 گروه درمانی
- تعیین و مقایسه میانگین مدت زمان بستری در بخش ICU در هريك از 3 گروه درمانی
- تعیین و مقایسه فراوانی مورتالیتی 30 روزه در هريك از 3 گروه درمانی
- تعیین و مقایسه میانگین مدت زمان تا منفی شدن تست سوآپ نازوفارینژیال در هريك از 3 گروه درمانی
- تعیین و مقایسه میانگین مدت زمان تا بهبود علائم بالینی در هريك از 3 گروه درمانی
- تعیین و مقایسه میانگین SOFA score در هريك از 3 گروه درمانی
- تعیین و مقایسه میانگین مدت زمان استفاده از تهویه مکانیکی در هريك از 3 گروه درمانی
  1. **هدف کاربردی (Practical Objective):**

معرفی یک روش درمانی جدید که باعث کاهش مورتالیتی و بهبود علائم در بیماران مبتلا به COVID-19 شود.

- 1. **فرضیات/سؤالات پژوهش (Hypothesis):**
- فراوانی عوارض جانبی در هريك از 3 گروه درمانی ذکر شده متفاوت است.
- میانگین سطح اشباع اکسیژن (Sat O2)در هريك از 3 گروه درمانی چقدر است؟
- میانگین نرخ تنفسی(RR) در هريك از 3 گروه درمانی چقدر است؟
- میانگین مدت زمان بستری در بیمارستان در هريك از 3 گروه درمانی چقدر است؟
- میانگین مدت زمان بستری در بخش ICU در هريك از 3 گروه درمانی چقدر است؟
- فراوانی مورتالیتی 30 روزه در هريك از 3 گروه درمانی متفاوت است.
- میانگین مدت زمان تا منفی شدن تست سوآپ نازوفارینژیال در هريك از 3 گروه درمانی چقدر است؟
- میانگین مدت زمان تا بهبود علائم بالینی در هريك از 3 گروه درمانی چقدر است؟
- میانگین SOFA score در هريك از 3 گروه درمانی چقدر است؟
- میانگین مدت زمان استفاده از تهویه مکانیکی در هريك از 3 گروه درمانی چقدر است؟

1. روش اجرای مطالعه (**Study Design**):

**3.1 روش اجرا (General):**

**نوع پژوهش:** کارآزمایی بالینی

**مکان و زمان پژوهش:** بیمارستان لقمان حکیم تهران در سال ۱۳۹۹

مطالعه حاضر یک کارآزمایی بالینی تصادفی شده است که در مقطع زمانی سال ۱۳۹۹ و پس از اخذ موافقت کمیته اخلاق دانشگاه علوم پزشکی شهید بهشتی بر روی بیماران مراجعه‌کننده دارای تست مثبت تایید کننده COVID-19 به مرکز آموزشی درمانی لقمان حکیم تهران انجام خواهد شد. بیماران با استفاده از روش تصادفی سازی Balance Block Randomization دریکی از سه گروه (گروه هیدروکسی کلروکین+کلترا + Interferon-β 1a و گروه هیدروکسی کلروکین+کلترا + Interferon-β 1bو گروه(درمان پایه) هیدروکسی کلروکین+کلترا) قرار می‌گیرند. برای این منظور از روش بلوک‌های تصادفی 3 تایی استفاده خواهیم نمود. به این شکل که 3 برگه کاغذی تهیه می‌نماییم (هر برگه مربوط به یک رژیم درمانی). روی هر برگه یکی از روش‌های درمانی مدنظر را می‌نویسیم. برگه‌ها را در کشو میز قرار می‌دهیم. با مراجعه‌ی هر یک از بیماران واجد شرایط و پس از تأیید پزشک در مورد معیارهای ورود به مطالعه،

یکی از برگه‌ها را به‌صورت تصادفی بیرون کشیده و به پرستار مسئول تهیه داروها تحویل داده‌شده و سپس داروی تهیه‌شده به بیمار جهت استفاده تحویل داده می‌شود. لازم به ذکر است برگه‌ بیرون کشیده شده تا زمانی که دو برگه دیگر بیرون کشیده نشده باشد به کشو برگردانده نخواهد شد. پس از بیرون کشیده شدن تصادفی 3 برگه، مجدداً برگه‌ها به کشو برگردانده شده و مجدداً عمل فوق برای 3 بیمار بعدی تا رسیدن به حجم نمونه موردنظر (n بیمار) ادامه داده خواهد شد.

- 1. **ارزیابی اثر بخشی (Efficacy Assessments):**
     1. **پیامدهای مطالعه (Study Outcome):**

**Primary Outcome:**

در این بخش زمان بهبود بالینی، معیار ما می باشد. که به شکل بهبود دو طبقه ای در مقیاس هفت طبقه ای منتشر شده توسط سازمان بهداشت جهانی یا ترخیص از بیمارستان، هر کدام که زودتر حاث شود، است.

**Secondary Outcomes:**

یکی از معیارهای پیامد ثانویه شامل مرگ و میر است؛ از اولین روز شرکت در مطالعه تا آخرین روز مطالعه است که روزی خواهد بود که کلیه بیماران حداقل یکی از نتایج زیر را داشته باشند: 1) بهبود دو طبقه ای در مقیاس هفت طبقه ای. 2) ترخیص از بیمارستان 3) مرگ.

از دیگر معیارهای پیامدهای ثانویه شامل: 1. بهبود SpO2 بین روز اول و آخر بستری، با استفاده از پالس اکسی متری؛ 2. مدت بستری از اولین روز شرکت در مطالعه تا تاریخ ترخیص از بیمارستان یا تاریخ فوت به هر علتی؛ 3. میزان

استفاده از تهویه مکانیکی جدید از زمان شرکت در مطالعه تا آخرین روز مطالعه. لطفا توجه داشته باشید که ما در تلاش هستیم نتایج ثانویه دیگری را اضافه کنیم و این بخش از پروتکل هنوز در حال تحول است.

- 1. **داروهای مطالعه (Study Drugs):**

هیدروکسی کلروکین:

400mg bid for first day then 200mg bid for 10 days

لوپیناویر/ریتوناویر:

400mg/100mg bid for 10-14 days

برای کسانی که نمیتوانند با دهان بخورند:

5-ml suspension bid for 10-14 days

اینترفرون بتا یک آ:

SC injection 44 micro-gram for 3 days (on days 1,3,6)

اینترفرون بتا یک ب:

SC injection 0.25 mg for 3 days (on days 1,3,6)

**3.4 تجزیه ‌و تحلیل داده‌ها (Statistical Methods):**

داده‌ها با استفاده از آمار توصیفی شامل میانگین، انحراف معیار، درصد و فراوانی و همچنین آمار تحلیلی شامل آزمون‌های کای اسکوئر، تی زوجی، ویلکاکسون، آنالیز واریانس ANOVA و آنالیز واریانس با اندازه‌گیری‌های مکرر و با استفاده از نرم‌افزار R نسخه 3.6.1 تجزیه‌وتحلیل می‌شوند. سطح معناداری روابط کمتر از 05/0 در نظر گرفته خواهد شد.

ابزار و روش جمع‌آوری داده‌ها

ابزار جمع‌آوری اطلاعات چک ‌لیست محقق ساخته است که در پیوست ۱ به انضمام درامده است.

1. **جمعیت مورد مطالعه (Study Population):**

4.1 شرکت کنندگان در مطالعه (**Participants**):

در ابتدا از بین بیماران مراجعه‌کننده به بیمارستان لقمان حکیم تهران در سال ۱۳۹۹ که تشخیص COVID-19 بر اساس شرح‌حال بیمار و معاینه بالینی و یا نتایج پاراکلینیک، توسط پزشک متخصص یا دستیار تخصصی برای آن‌ها گذاشته‌شده، با در نظر گرفتن معیارهای ورود، بیماران واجد شرایط مطالعه انتخاب می‌شوند. پس از انتخاب بیماران به‌عنوان نمونه اولیه به بیماران جهت شرکت در این مطالعه توضیحات لازم داده خواهد شد و به آن‌ها برای شرکت در این مطالعه حق انتخاب داده خواهد شد. در صورت موافقت به مشارکت در این مطالعه بیماران وارد یک مطالعه‌ی تصادفی شده می‌شوند و به‌صورت تصادفی بیماران را به سه گروه تقسیم می‌کنیم، بیماران روزی n بار (با فواصل n ساعت) این دارو ها را به صورت تزریقی مورد استفاده قرار می دهند. نحوه تصادفی سازی افراد به سه گروه مورد مطالعه به‌منظور بالانس بوده و تعداد افراد در سه گروه مطالعه از نوع تصادفی بلوکه خواهد بود و بلوک‌هایی 3 تایی تشکیل خواهد شد.

جامعه پژوهش: کلیه بیماران دارای تست مثبت تایید کننده COVID-19 و دارای معیارهای ورود به مطالعه مراجعه‌کننده به بیمارستان لقمان حکیم تهران

**روش انتخاب افراد:** به‌صورت در دسترس از تمام بیماران مراجعه‌کننده بیمارستان لقمان حکیم تهران و دارای معیارهای ورود به مطالعه، از زمان شروع مطالعه تا پایان سال 1400

محاسبه اندازه نمونه:

طبق مطالعه لاتوش و همکاران در سال 2004 مطالعه برای پاور 80% و هازارد ریشیو 2.5 محاسبه خواهد شد. (با فرض بهبود یافتن 80% بیماران)

- 1. **معیارهای ورود (Inclusion Criteria):**
- بیماران با سن ≥ 18 سال.
- بیماران تایید شده کووید-19 ) با روش RT-PCR (
- حداقل یکی از موارد زیر:

دمای پیشانی ≥ 37.8 درجه سانتیگراد، سرفه، گلو درد، احتقان بینی، میالژی، سردرد یا خستگی در هنگام پذیرش

- SPO2 ≤ 93% OR respiratory rate ≥ 24
- مدت زمان شروع علائم≤ 14 روز.
  1. **معیارهای خروج (Exclusion Criteria):**
- عدم رضایت برای شرکت در مطالعه
- بیماران مبتلا به QT یا PR طولانی، بلوک درجه دوم یا سوم قلب یا آریتمی های قلبی، از جمله torsade de pointes
- بیمارانی که داروهایی مصرف می کنند که به طور بالقوه با هیدروکسی کلروکین+کلترا ، Interferon-β 1a، Interferon-β 1b تداخل دارند
- زنان باردار یا شیردهی
- در 5 سال گذشته الکل یا مواد مخدر را داشته باشد
- سطح آلانین آمینوترانسفراز (ALT)> 5 برابر بالاتر از حد نرمال که در آزمایشگاه بیمارستان تست شده است
  1. **ریزش مطالعه (Drop Out):**

در صورت بروز آلرژی به دارو یا ترک بیمار از اورژانس و یا انصراف بیمار از همکاری به‌عنوان ریزش مطالعه در نظر گرفته خواهد شد.

1. **محدودیت ها (Restrictions):**

محدودیت‌های این مطالعه شامل موارد زیر خواهند بود:

۱. عدم رضایت شرکت در طرح

۲. عدم پاسخ به درمان

۳. خروج بیماران از مطالعه

4. عدم دسترسی به داروهای مذکور

5. در دسترس نبودن تعداد بیماران مورد نیاز

1. **ملاحظات اخلاقی (Ethical Considerations):**

- قبل از اجرای طرح تأییدیه کمیته اخلاق و شورای پژوهشی دانشگاه علوم پزشکی شهید بهشتی اخذ خواهد گردید
- تمامی مراحل تحقیق در IRCT ثبت خواهد گردید.
- از کلیه بیماران و یا قیم قانونی آن‌ها قبل از ورود به مطالعه رضایت‌نامه مکتوب اخذ می‌گردد.
- اطلاعات کامل و شفاف در ارتباط با مراحل تحقیق در اختیار بیماران و یا قیم قانونی آن‌ها قرار خواهیم داد.
- در هر مرحله از تحقیق که بیماران قصد خروج از مطالعه را داشته باشند این اجازه به آن‌ها داده می‌شود.
- پرونده کلیه بیماران به‌صورت کامل محفوظ مانده و شرط امانت رعایت می‌شود.
- جهت هیچ مرحله‌ای از تحقیق از بیماران وجهی دریافت نخواهد شد.

1. **منابع (Refernces):**

1. Hageman JR. The Coronavirus Disease 2019 (COVID-19). Pediatr Ann [Internet]. 2020;49(3):e99–100. Available from: http://www.ncbi.nlm.nih.gov/pubmed/32155273

2. Lu R, Zhao X, Li J, Niu P, Yang B, Wu H, et al. Genomic characterisation and epidemiology of 2019 novel coronavirus: implications for virus origins and receptor binding. Lancet (London, England). 2020 Feb;395(10224):565–74.

3. Chen Y, Liu Q, Guo D. Emerging coronaviruses: Genome structure, replication, and pathogenesis. J Med Virol. 2020 Apr;92(4):418–23.

4. Spiegel M, Pichlmair A, Mühlberger E, Haller O, Weber F. The antiviral effect of interferon-beta against SARS-Coronavirus is not mediated by MxA protein. J Clin Virol. 2004;30(3):211–3.

5. Channappanavar R, Perlman S. Pathogenic human coronavirus infections: causes and consequences of cytokine storm and immunopathology. Semin Immunopathol. 2017 Jul;39(5):529–39.

6. Zhou Y, Hou Y, Shen J, Huang Y, Martin W, Cheng F. Network-based Drug Repurposing for Human Coronavirus. medRxiv [Internet]. 2020 Jan 1;2020.02.03.20020263. Available from: http://medrxiv.org/content/early/2020/02/05/2020.02.03.20020263.abstract

7. Mosaddeghi P, Negahdaripour M, Farahmandnejad M, Taghipour MJ, Moghadami M, Nezafat N, et al. Therapeutic approaches for COVID-19 based on the dynamics of interferon- mediated immune responses. 2020;(March).

8. Cinatl J, Morgenstern B, Bauer G, Chandra P, Rabenau H, Doerr HW. Treatment of SARS with human interferons. Lancet. 2003;362(9380):293–4.

9. Lokugamage KG, Schindewolf C, Menachery VD. SARS-CoV-2 sensitive to type I interferon pretreatment. bioRxiv [Internet]. 2020 Jan 1;2020.03.07.982264. Available from: http://biorxiv.org/content/early/2020/03/09/2020.03.07.982264.abstract

10. Hensley LE, Fritz EA, Jahrling PB, Karp CL, Huggins JW, Geisbert TW. Interferon-β 1a and SARS Coronavirus Replication. Emerg Infect Dis. 2004;10(2):317–9.
